# Trends in malaria morbidity among health care-seeking children under age five in Mopti and Sévaré, Mali between 1998 and 2006

**DOI:** 10.1186/1475-2875-9-319

**Published:** 2010-11-11

**Authors:** Alyson Rose-Wood, Seydou Doumbia, Bouyagui Traoré, Marcia C Castro

**Affiliations:** 1 Department of Global Health and Population, Harvard School of Public Health, Harvard University, Boston, Massachusetts, USA; 2 Department of Public Health and Malaria Research and Training Center, Faculté de Médecine de Pharmacie et d'Odonto-Stomatologie (FMPOS), Bamako, Mali; 3 Direction Nationale de la Santé, Ministry of Health, Bamako, Mali

## Abstract

**Background:**

In Mali, malaria is the leading cause of death and the primary cause of outpatient visits for children under five. The twin towns of Mopti and Sévaré have historically had high under-five mortality. This paper investigates the changing malaria burden in children under five in these two towns for the years 1998-2006, and the likely contribution of previous interventions aimed at reducing malaria.

**Methods:**

A retrospective analysis of daily outpatient consultation records from urban community health centres (CSCOMs) located in Mopti and Sévaré for the years 1998-2006 was conducted. Risk factors for a diagnosis of presumptive malaria, using logistic regression and trends in presumptive malaria diagnostic rates, were assessed using multilevel analysis.

**Results:**

Between 1998-2006, presumptive malaria accounted for 33.8% of all recorded consultation diagnoses (10,123 out of 29,915). The monthly presumptive malaria diagnostic rate for children under five decreased by 66% (average of 8 diagnoses per month per 1,000 children in 1998 to 2.7 diagnoses per month in 2006). The multi-level analysis related 37% of this decrease to the distribution of bed net treatment kits initiated in May of 2001. Children of the Fulani (Peuhl) ethnicity had significantly lower odds of a presumptive malaria diagnosis when compared to children of other ethnic groups.

**Conclusions:**

Presumptive malaria diagnostic rates have decreased between 1998-2006 among health care-seeking children under five in Mopti and Sévaré. A bed net treatment kit intervention conducted in 2001 is likely to have contributed to this decline. The results corroborate previous findings that suggest that the Fulani ethnicity is protective against malaria. The findings are useful to encourage dialogue around the urban malaria situation in Mali, particularly in the context of achieving the target of reducing malaria morbidity in children younger than five by 50% by 2011 as compared to levels in 2000.

## Background

Malaria is a major cause of childhood morbidity and mortality in sub-Saharan Africa. In 2006, 86% of the estimated 247 million malaria cases occurred in that region, causing 801,000 deaths (85% among children under age five) [1]. Efforts to address this burden have been undertaken (e.g., Global Fund, President's Malaria Initiative, and the recent call for malaria elimination/eradication) and continue to expand [2-5]. Significant declines are starting to be observed, as those recently reported for Kenya and The Gambia [6,7].

In Mali, malaria is the leading cause of death and of outpatient visits for children under five [8]. Nationally, 76% of deaths attributed to malaria occur in children under five [9]. With 32% of the population living in cities, Mali is less urbanized than other countries in West Africa [9-11]. However, its urban areas are growing rapidly (approximately 4.8% per year), a result of both natural increase and rural-urban migration due to successive droughts between 1973-1997 and 2001-2002 [12,13]. It is possible that this migration has concurrently seen an increase in malaria, since rural migrants are more likely to be infected with malaria [13-16]. The lack of good surveillance, among other issues, has prohibited the Malian Ministry of Health (MoH) from implementing systematic interventions targeted to urban settings [17].

Mopti Region continuously posts the poorest health indicators in Mali, with mortality rates for children under age five consistently higher than the national average [8]. A 1985 household demographic and health survey in Mopti and Sévaré found that these two towns had an exceptionally high under-five mortality rate (U5MR), with between 30% and 50% of children dying before the age of five [18]. Although the U5MR continues to be extremely high (227 per 1,000 live births in 2006), these two towns are not considered a zone with high malaria risk by the MoH, and therefore receive limited support for control [8,9,18]. Yet, approximately one third of all children under age five who reported to a health facility between 1998-2006 were diagnosed as a presumptive malaria case.

In 1987, the Government of Mali implemented a decentralization of the health care system down to the regional level, followed by the creation of the National Malaria Control Programme in 1993 [9]. In 1999, the Government of Mali committed itself to the Roll-Back Malaria Initiative and held a National Forum on Malaria in Mopti [9]. Following the Forum, the Mopti Regional Health Directorate (DRS) implemented several malaria control interventions in Mopti and Sévaré, including: training of 18 microscopists in malaria diagnosis (April, 2000) [Barry A., personal communication, February 25, 2009]; distribution of free bed net treatment kits through the urban community health centers (CSCOM) and the private sector (May-September, 2001) [Barry A., personal communication, February 25, 2009] [19]; and a social marketing campaign supported by the U.S. Agency for International Development (USAID) (October 2003 to December 2006) [20,21]. Although all CSCOMs keep records of outpatient cases by cause, little is known about trends in diagnosed malaria.

This paper aims to use daily consultation records from all CSCOMs located in Mopti and Sévaré to conduct a retrospective analysis of trends in malaria morbidity in children under age five between 1998-2006, and to evaluate the potential impact of malaria interventions implemented throughout the period. For the purposes of this paper, the terms 'presumptive malaria diagnosis' and 'malaria consultation' are used interchangeably.

## Methods

### Study area

**Figure 1:**
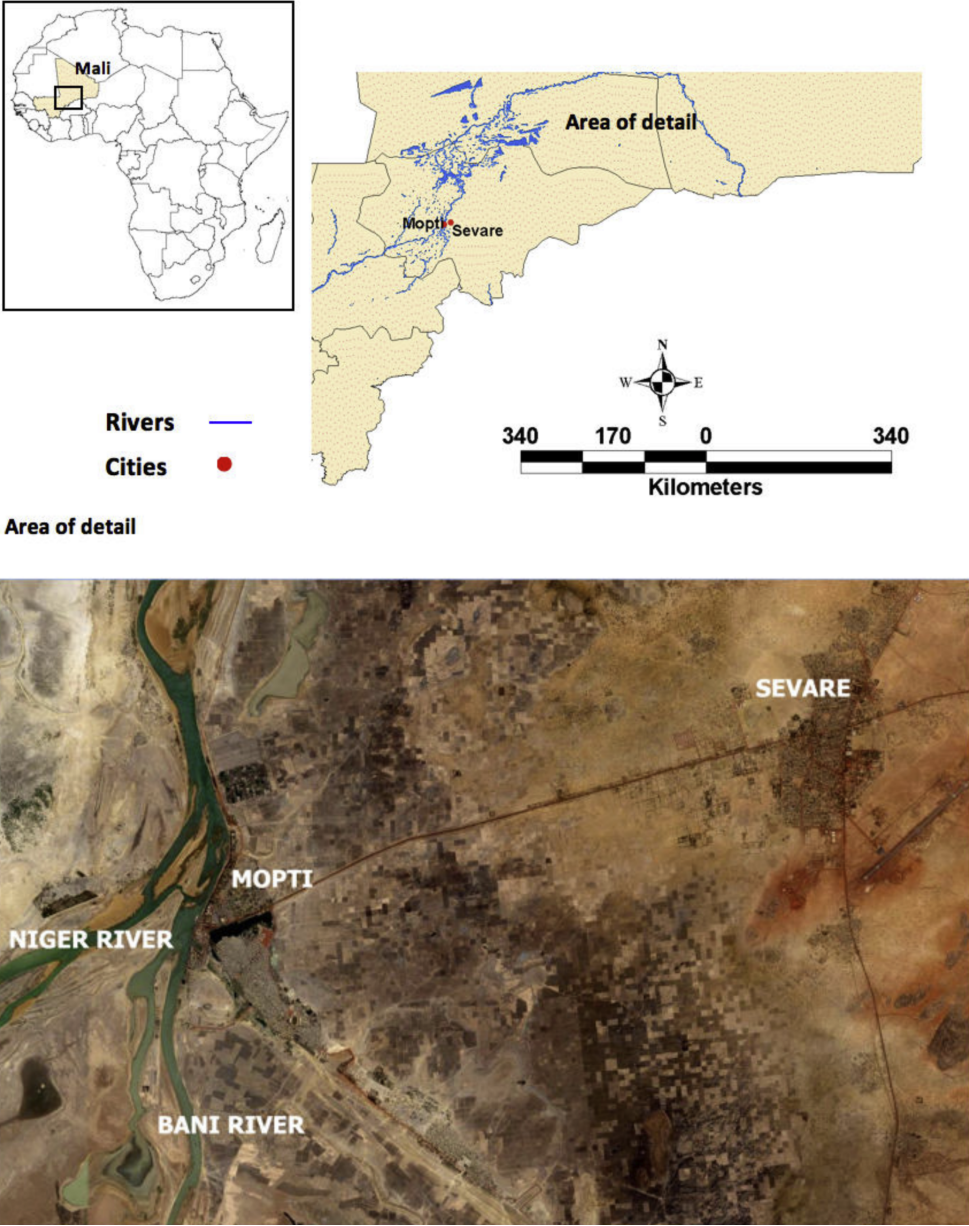
**Geographical location and aerial view of the study area**. The aerial view shows the study area - Mopti and Sévaré, in Mali - extracted from an IKONOS satellite image (captured in April 2004, and provided by the GeoEye Foundation). The area is located within the bifurcated Niger and Bani Rivers.

The twin towns of Mopti and Sévaré had an estimated population of 71,000 and 49,000, respectively, in 2006 [22]. Separated by 11 km, they are located in Mopti Region, approximately 750 km north-east of Mali's capital, in the Niger River Inland Delta (Figure 1) [23]. The delta is characterized by seasonal floods (September to January) that favour the proliferation of water habitats ideal for mosquito breeding [24]. The area has a typical Sahelian climate with annual rainfall between 200-500 mm, concentrated in a single rainy season from June to September and followed by a long dry season of 8-9 months [25].

The predominant malaria vector is *Anopheles gambiae s.l*. At peak transmission during the rainy season, the entomologic inoculation rate (EIR) can reach 41 infective bites per person per month [25]. *Plasmodium falciparum *is the predominant type of parasite (>95%) [25]. The area is considered endemic for malaria, marked by stable and low transmission with peaks during the rainy season [9].

**Figure 2:**
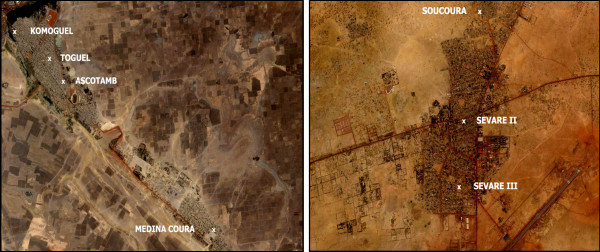
**Geographic location of community health centers (CSCOMs), Mopti and Sévaré, Mali**. All seven urban CSCOMs located in Mopti (left) and Sévaré (right) are shown utilizing an IKONOS satellite imagery (captured in April 2004, and provided by the GeoEye Foundation) as reference. The catchment population of each CSCOM is roughly comprised by nearby residents.

Each subdivision of the towns of Mopti and Sévaré has a CSCOM, typically run by a nurse or a physician, and controlled by a community health association. CSCOMs serve as the primary level of care, providing referrals to the only hospital in the region. Each CSCOM has a designated catchment population, and is roughly located in the center of the community it serves [26]. A total of seven CSCOMs are located in the study area: four in Mopti and three in Sévaré (Figure 2). Five of these CSCOMs were already established in 1998, while two, Sévaré III and Toguel, were opened in 2004 in response to the increased demand for health services due to rapid urbanization. With seven CSCOMs, the populations of Mopti and Sévaré have high access to primary health care relative to other urban areas of Mali.

### Health facility data

In 1998, the DRS launched an effort to improve CSCOM records, including random inspections of consultation registries, along with the implementation of a quarterly reporting system [Barry A., personal communication, February 25, 2009]. In this paper, monthly outpatient consultations recorded between 1998-2006 in each CSCOM in Mopti and Sévaré were used. Since CSCOMs do not have laboratory facilities and only 0·8% of all presumptive malaria cases were referred to the hospital-based laboratory, these data refer to presumed malaria cases, based on a temperature of 37·5°C or higher and/or a combination of common symptoms of malaria. This diagnostic procedure tends to overestimate the number of confirmed malaria cases [27-30]. However, since there was no change in CSCOMs' malaria diagnostic capabilities between 1998-2006, it was assumed that eventual biases towards a malaria diagnosis were consistent throughout the study period.

All information was collected between June and August of 2008, recorded in a database, and included age (in months) of each child under five, ethnicity, sex, diagnosis, and CSCOM name (Table 1). Quarterly CSCOM reports were used to gather information on the number of doctors and nurses effectively working at each CSCOM (assumed to remain constant during each three-month period), and the annual estimated catchment population of each CSCOM. Annual population estimates of each town were obtained from the Statistics Office of the DRS.

**Table 1:** Variables used in the retrospective analysis of trends in child morbidity in Mopti and Sévaré, Mali between 1998 and 2006

Variable	Source	Description	Categories
**Demographic**			

Age	Daily consultation records, by CSCOM	Age of outpatient child in months	(1) 0-5 months, (2) 6-17 months, (3) 18-59 months
Sex	Daily consultation records, by CSCOM	Sex of outpatient child	(0) Male, (1) Female
Ethnicity	Daily consultation records, by CSCOM	Ethnic group of outpatient child	(1) Peuhl/Fulani, (2) Sonhay, (3) Bambara, (4) Bozo, (5) Dogon, (6) Other (Sarakole, Malinke, Touareg, Arab, Bella, Samogo, Mossi, Senoufo, Minianka, Woloffe, Dafin, Hawsa)

**Malaria control**			

Bednet treatment kit distribution	Mopti Regional Health Directorate	Bednet treatment kits were distributed through CSCOMs and for sale through the private sector beginning in May 2001.	(0) Pre-distribution, (2) Post-distribution
National bednet social marketing campaign	Mopti Regional Health Directorate; Population Services International (PSI)	Public service announcements commenced October 2003.	(0) Pre-social marketing campaign, (1) Post-social marketing campaign

**Climate and local ecology**			

Annual rainfall	African Data Dissemination Service/Famine Early Warning System Meteosat satellite-based rainfall estimates	Annual standard deviation values computed from the 9-year (1998-2006) mean.	(1) greater than 2 SD below the mean, (2) within 2 SD of the mean, (3) greater than 2 SD above the mean
Monthly rainfall	African Data Dissemination Service/Famine Early Warning System Meteosat satellite-based rainfall estimates	Rainfall (mm) estimates based on the sum of 3 dekads per month.	(1) <15 mm, (2) ≥15 mm and ≤90 mm, (3) >90 mm
Lagged monthly rainfall		One-month lagged rainfall.	(1) <15 mm, (2) ≥15 mm and ≤90 mm, (3) >90 mm
CSCOM water proximity during the non-flood period	IKONOS satellite imagery (April 2004)	Shortest distance measured from each CSCOM to the permanent water body during the non-flood period (April).	Continuous variable in meters (mean = 786.26 meters)
CSCOM water proximity during delta flooding	IKONOS satellite imagery (November 2004)	Shortest distance measured from each CSCOM to the flooded river/lake during the flood period (November).	Continuous variable in meters (mean = 391.92 meters)

**Health care resources**			

CSCOM	Daily consultation records	Names of each CSCOM	(1) Ascotamb, (2) Toguel, (3) Soucoura, (4) Sévaré II, (5) Sévaré III, (6) Komoguel, (7) Medina Coura
Doctors	CSCOM Quarterly Reports, Mopti Regional Health Directorate	Doctors in each CSCOM per 10,000 people by month and year (# of doctors/annual CSCOM catchment population)	Continuous variable
Nurses	CSCOM Quarterly Reports, Mopti Regional Health Directorate	Nurses in each CSCOM per 10,000 people by month and year (# of nurses/annual CSCOM catchment population)	Continuous variable

The proportion of the population served by individual CSCOMs in each year of the study period was calculated by dividing the estimated population in both towns by the estimated catchment population. Since the latter was not estimated by age groups, monthly malaria consultation rates per 1,000 children under five for each CSCOM were calculated utilizing as denominators the product of the annual catchment population by the national estimated proportion of children under five for each year.

Finally, information was gathered on malaria control interventions implemented during 1998-2006 through personal communication with the Director of Statistics at the DRS, and through a literature search.

### Rainfall data

A monthly time series of rainfall from satellite-based estimates provided by the African Data Dissemination Service (ADDS) [31] was assembled. Estimates are available by dekads (periods of roughly ten days) with a 2 km spatial resolution. A total of 1,296 files, each comprising 270,000 pixels, were analyzed in ArcGIS 9.2 (ESRI, Redlands, CA, USA) in order to extract rainfall data for the study area. These data were summarized by year and month (Table 1). To capture anomalies, annual rainfall was summarized as the number of standard deviations from the study period mean. Categories for monthly rainfall were selected based on the 25^th ^and 75^th ^percentiles. The effect of rainfall on *Anopheles *density was expected to be lagged in time, although the exact time lag is dependent on temperature and may vary locally [32-37]. In this paper, a one month lagged rainfall variable was tested.

To accommodate the annual flooding pattern of the inland delta, high-resolution imagery was utilized to calculate the distance from each CSCOM to the nearest body of water in the delta. The distance was measured for non-flood (February-August) and flood (September-January) periods (Table 1). Pan-sharpened IKONOS satellite images (1 m of spatial resolution) were obtained through The GeoEye Foundation for April and November of 2004 (a year of normal rainfall). The images were analysed in ArcGIS 9.2. All calculations were performed in STATA 10.0 (Stata Corp., College Station, TX, USA).

### Statistical analysis

Potential impacts of demographic and environmental variables on malaria diagnoses were investigated with logistic regression models. The response variable indicated if malaria was diagnosed for each child under age five who sought care in the CSCOM. The covariates included in the model were: ethnicity, age, CSCOM, monthly rainfall, sex, and year (Table 1). Age categories (0-5 months, 6-17 months, and 18-59 months) are those used in previous studies conducted in Mopti Region [18]. The fit of the model was assessed using the Pearson Chi-Square Goodness-of-Fit test.

Trends in malaria consultation rates over time were appraised with a multilevel model for change. Because the data utilized in this paper did not follow individual children over time, the CSCOMs were used as the unit of analysis. For each CSCOM, predictor variables were summarized by month. Individual characteristics were aggregated by month: mean age of all the children; proportion of children who were male; and proportion of children from Bozo and Dogon ethnicities (the principal ethnic groups).

The multilevel model for change was fitted using a generalized least squares method, and included components at two levels: a level-1 sub-model that described how the monthly malaria consultation rate in each CSCOM changed over time, and a level-2 sub-model that described how these changes varied across CSCOMs. The assumption was that the shape of the hypothesized individual monthly CSCOM malaria consultation rate change trajectory was linear. Assumptions about the error distributions specified univariate normality at level-1 and bivariate normality at level-2. The fit of the model was assessed using the Akaike Information Criterion (AIC) [38].

### Human subjects

This study was exempted of human subjects by the Harvard School of Public Health Institutional Review Board (protocol # P16245-101) and by the Ethics Committee of the Faculty of Medicine, Pharmacy, and Odontostomatology, University of Bamako (protocol # 61/FMPOS).

### Role of the funding source

The sponsor of the study had no role in the design, data collection, data analysis, data interpretation, or writing of the report. The authors had full access to all the data in the study and had final responsibility for the decision to submit for publication.

## Results

### Characteristics of the population

**Figure 3:**
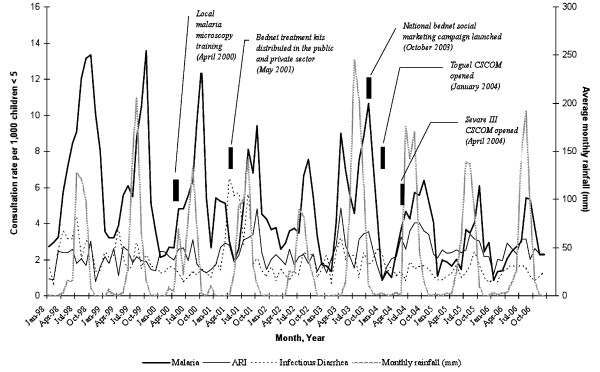
**Time-series of monthly incidence rates for malaria, acute respiratory infection, and infectious diarrhea between 1998 and 2006 plotted against monthly rainfall**. Monthly rainfall (mm) plotted against malaria, acute respiratory infection (upper and lower combined), and infectious diarrhea monthly consultation rate per 1,000 children under age five in Sévaré and Mopti, Mali from January 1998 through December 2006. Key interventions to reduce malaria incidence, and the expansion of CSCOMs in the study area are indicated in the graph. Source: Monthly consultation registries and ADDs estimates.

Monthly consultation records for 51 independent diagnoses were summarized into 14 disease categories using version 10 of the International Classification of Diseases coding. Of the total observations (n = 30,198), 283 were incomplete. As there was not a pattern in missing data, all incomplete observations were removed from the analysis. Also, seven of the 606 total monthly records were missing (Table 2). Malaria inflicted the most morbidity in children under age five (33·8%) followed by upper and lower acute respiratory infections (ARI) (17·7%), and infectious diarrhea (14·0%). Combined, these three disease categories accounted for 65·5% of all diagnoses (Figure 3). A total of 10,123 children under age five were diagnosed with malaria during the study period. The time series of monthly presumptive malaria consultation frequency showed a strong decreasing secular trend between 1998 (15·6%) and 2006 (7·4%), in contrast to the more stable frequencies of both ARI and infectious diarrhea over this same time period (Figure 3).

**Table 2:** Summary of missing data on CSCOM consultation records

CSCOM	Town	Population (2006)	Study period	Missing year	Missing months	% Missing
Sevare II	Sévaré	23,288	01/1998-12/2006	1998 (1)	1	0·0017
Sevare III*	Sévaré	11,904	04/2004-12/2006	-	0	0·0000
Soucoura	Sévaré	14,980	01/1998-12/2006	-	0	0·0000
Ascotamb	Mopti	27,633	01/1998-12/2006	1998 (2), 2003 (1)	3	0·0050
Komoguel I	Mopti	23,288	01/1998-12/2006	-	0	0·0000
Medina Coura	Mopti	6,870	01/1998-12/2006	1998 (3)	3	0·0050
Toguel*	Mopti	13,640	04/2004-12/2006	-	0	0·0000

Total		121,603	606 months	-	7	0·0116

*CSCOM opened during time-series period

The projected number of individuals (2006) served by each community health center (CSCOM) service area within the towns of Mopti and Sévaré is tabulated under the Population heading. Potential consultation records are listed under Study period. Unavailable CSCOM clinical records appear under Missing year - the number of missing monthly records for each year is listed in parenthesis. These are totaled under Missing months and expressed as percentages from the total number of possible monthly records (across all CSCOM service areas) under the % Missing heading. The total projected population (2006) for Mopti and Sévaré is 121,603 individuals.

Annually, malaria consultations peaked during August-October, which coincides with the end of the rainy season. Dogon was the principal ethnic group (33·2%), followed by Bozo (24·5%). The Fulani (Peuhl) ethnic group had the lowest percentage of malaria consultations (6·4%).

### Risk factors for a diagnosis of presumptive malaria

Univariate tests on malaria interventions indicated that only the distribution of bed net treatment kits was a significant predictor of both the monthly malaria consultation rate (p < 0·001) and the change in the monthly malaria consultation rate over time (p < 0·001). The same procedure was undertaken for the control variables, and those selected for inclusion in the model were: age, sex, ethnicity, annual rainfall, monthly rainfall, lagged monthly rainfall, CSCOM water proximity during the non-flood period, CSCOM water proximity during delta flooding, CSCOM, nurses, and doctors.

Results of the logistic regression model are shown in Table 3. Overall, the odds of a diagnosis of malaria decreased between 1998-2006: compared to 1998, the odds increased by 71% (95% CI 1·632-1·792) in 1999, but were reduced by 19% (95% CI 0·725-0·903) in 2006. Significant declines in the odds of a diagnosis began in 2004. This temporal pattern supports the use of the multilevel model to assess changes in the monthly malaria consultation rate over time.

**Table 3:** Logistic regression analysis of factors influencing the diagnosis of presumptive malaria among children younger than 5 years, Mopti and Sévaré, 1998-2006

Covariate	Odds Ratio (OR)	OR 95% CI	P-value
CSCOM			

Ascotamb	--	--	--
Toguel	1·236	1·058-1·443	<0·0001
Sévaré II	0·721	0·676-0·768	<0·0001
Sévaré III	0·784	0·776-0·852	0·0221
Soucoura	0·718	0·669-0·751	0·0316
Komoguel	0·793	0·740-0·849	<0·0001
Medina Coura	0·698	0·639-0·763	<0·0001

YEAR			

1998	--	--	--
1999	1·707	1·632-1·792	<0·0001
2000	1·647	1·576-1·726	<0·0001
2001	1·841	1·757-1·934	0·0014
2002	1·205	1·086-1·337	0·0005
2003	1·022	0·921-1·134	0·4838
2004	0·858	0·773-0·952	0·0195
2005	0·865	0·781-0·959	<0·0001
2006	0·810	0·725-0·903	0·0024

MONTHLY RAINFALL			

<15 mm	--	--	--
≥15 mm and ≤90 mm	1·704	1·665-1·745	<0·0001
>90 mm	1·846	1·764-1·901	<0·0001

SEX			

Male	--	--	--
Female	0·946	0·929-1·025	0·3754

ETHNICITY			

Fulani/Peuhl	--	--	--
Sonhay	1·630	1·506-1·765	<0·0001
Bambara	1·573	1·446-1·711	<0·0001
Bozo	1·671	1·531-1·824	<0·0001
Dogon	2·358	2·161-2·574	<0·0001
Other	1·618	1·496-1·749	<0·0001

AGE			

0-5 months	--	--	--
6-17 months	1·034	0·951-1·123	0·4456
18-59 months	1·285	1·187-1·391	<0·0001

χ_^2^_	50·2		

Increases in rainfall consistently led to more presumptive malaria diagnoses. Compared to months with <15 mm of rainfall, the odds of a malaria diagnosis increased by 70% in months that had precipitation levels between 15 mm and 90 mm, and by 85% in months that had more than 90 mm of rain. Children aged 18-59 months had 1·29 times the odds of a malaria diagnosis than children age 0-5 months. Finally, the Pearson Chi-Square Goodness-of-Fit test was significant (p < 0·0001).

### Trends in malaria morbidity

Results of the multilevel model are presented in Table 4: Model A described the variation in the monthly malaria consultation rate across CSCOMs without regard to time or other predictors; Model B added time variables; and Models C and D included several predictors.

**Table 4:** Multilevel models for malaria consultation rate change across community health centers and time, Mopti and Sévaré, 1998-2006

	Model A	Model B	Model C	Model D
**Fixed Effects**				

Intercept	4·973**** (0·957)	6·720**** (1·691)	10·127*** (3·587)	9·824*** (3·659)

Bednet treatment kits			-8·146**** (1·649)	-8·163**** (1·654)

1999			-2·772* (1·491)	-2·794* (1·496)

2000			-7·192*** (2·246)	-7·227*** (2·247)

2001			-9·006*** (3·101)	-9·107*** (3·113)

2002			-13·223*** (4·229)	-13·255*** (4·231)

2003			-15·858 (14·984)	-15·978 (14·876)

2004			-17·922*** (5·981)	-18·117** (8·736)

2005			-23·298*** (6·921)	-23·509*** (6·941)

2006			-27·765**** (7·898)	-28·023**** (7·929)

Monthly rainfall			0·374 (0·359)	0·348 (0·361)

Lagged monthly rainfall				0·154* (0·092)

CSCOM water proximity during delta flooding			0·003 (0·034)	0·011* (0·006)

CSCOM water proximity during non-flooding			-0·0003 (0·0004)	-0·001* (0·0006)

Intercept		-0·027* (0·016)	-0·204** (0·096)	-0·231** (0·110)

Bednet treatment kits			-0·164**** (0·037)	-0·162**** (0·036)

**Variance Components**				

Within-CSCOM	38·615** (19·652)	36·096** (18·403)	32·175** (12·295)	32·129** (12·249)

In initial status	5·893* (3·163)	14·320* (7·698)	2·656* (1·389)	2·651* (1·386)

In rate of change		0·001 (0·006)	0·001 (0·015)	0·001 (0·015)

Covariance		-0·105 (0·086)	-0·033 (0·199)	-0·033 (0·197)

**Goodness-of-Fit**				

AIC	3660·969	3644·288	3631·620	3629·451

* p < .10; ** p < .05; *** p < .01; **** p < .001; standard deviation marked in ()

Models A and B describe the variation in the malaria consultation rate over time between January 1998 and December 2006 without controlling for any predictors. Models C and D predict the malaria consultation rate change between January 1998 and December 2006 controlling for health care resource variables (CSCOM, nurses, doctors) and demographic variables (male, Bozo, Dogon, mean age).

The average monthly malaria consultation rate across all CSCOMs irrespective of time was 4·97, and 13·2% (5·893/(5·893+38·615)) of the total variation in malaria consultation rates was attributable to variation between CSCOMs (Table 4, Model A). Before controlling for demographic and environmental variables, the average CSCOM had an initial monthly malaria consultation rate of 6·72, and a consultation rate change of -0·027 per month (Table 4, Model B). Also, 6·5% ((38·615-36·096)/38·615) of the within-CSCOM variation in the malaria consultation rate was associated with time, and 28·4% (14·320/(14·320+36·096)) of the total variation in malaria consultation rates was attributable to variation between CSCOMs over time.

Controlling for demographic variables and health care resources, bed net distribution had a significant effect on the monthly malaria consultation rate (Table 4, Model C). Prior to bed net distribution, the average CSCOM had an initial malaria consultation rate of 10·13, and a consultation rate change of -0·204 per month. After the bed net treatment kit distribution, the average CSCOM had an initial consultation rate of 1·98 and a consultation rate change of -0·368 per month. These results suggest that the bed net treatment kit distribution may have reduced the malaria consultation rates over time. Post-intervention, there was a 45% reduction in the monthly malaria consultation rate. The covariates included in this model explained 81·5% ((14·32-2·656)/14·32) of the variation in the malaria consultation rate between CSCOMs and 10·9% ((36·096-32·175)/36·096) of the variation within-CSCOMs.

Regarding rainfall, Model D suggested that one-month lagged rainfall, unlike monthly rainfall, was a significant predictor of the malaria consultation rate (p = 0·092). Accounting for lagged rainfall, the slope of the monthly malaria consultation rate change trajectory after the bed net treatment kit distribution was 37% lower than prior to the intervention. The AIC suggested that Model D provided a better fit compared to Model C. Standardized residuals fell within +/- 2 standard deviations of their mean, suggesting that the normality assumption was not violated.

## Discussion

The results suggest a 66% secular decline in the presumptive malaria consultation rate between 1998-2006 in Mopti and Sévaré, significantly associated with the distribution of bed net treatment kits. In contrast, the bed net social marketing campaign did not reveal significant effects. Substantial variation in the monthly malaria consultation rate was found both between and within CSCOMs, and over time, and corroborated preliminary findings that the Fulani (Peuhl) ethnicity has a protective effect on malaria.

The findings are consistent with recent reports of malaria declines observed in Kenya and The Gambia [6,7]. Similar to these studies, we do not identify a specific cause for the decline in the malaria consultation rate. Several variables, however, did show a significant association with a diagnosis of malaria, including ethnicity, age of the child, monthly rainfall, and lagged monthly rainfall. Of the three malaria interventions implemented in Mopti and Sévaré between 1998-2006, only the distribution of bed net treatment kits was a significant predictor of both the initial malaria consultation rate and the estimated rate of change in the consultation rate over time.

Regarding ethnicity, children from the Fulani (Peuhl) group had significantly lower odds of a diagnosis of malaria when compared to every other ethnicity. Decreased risk for malaria among the Fulani has been reported in studies in Mali and Burkina Faso, despite few cultural and socioeconomic differences, and similar levels of exposure as measured by the EIR [39-43]. Compared to sympatric groups, the Fulani often have a lower parasite rate, are less affected by malaria, and have higher levels of anti-malaria antibodies [39-41]. However, the mechanisms that confer this protection remain largely unknown.

The age of the child is an important consideration in discussions of malaria diagnosis. This study found that children aged 18-59 months were at increased risk (OR = 1·285; 95% CI: 1·187-1·391) for a diagnosis of malaria as compared to infants (0-5 months). This finding is consistent with a recent study in Kenya that showed that when there is reduced exposure to infection following a decline in malaria transmission, there is a corresponding increase in the mean age of children with malaria [6]. In addition, since Mopti and Sévaré are urban areas of low malaria transmission, it is possible that immunity to malaria is not reached until children are older, which emphasizes the need for age-specific interventions [16].

Monthly malaria consultation rates decreased by 37% after the bed net treatment kit distribution. The likely success of this intervention might be explained by the long history of bed net ownership in Mopti and Sévaré, coupled with positive perceptions of the insecticide used to treat them [44]. A 2003 baseline NetMark (a USAID-funded partnership) survey conducted in Mopti found that 51% of those who reported owning a treated bed net had received the net treatment for free from a CSCOM [44]. Also, the survey reported high levels of bed net usage in Mopti; 98.8% of sampled households had used a bed net the previous night, including 80% of children under five [44]. In contrast, the 2003 bed net social marketing campaign did not significantly impact the malaria consultation rate. It is possible that the campaign, targeting the proper use of bed nets, had little influence on bed net usage in the two towns due to the historic high levels of net ownership and usage [9,20,44].

According to the 2006 DHS, 50% of households in Mali have at least one mosquito net, but only one in five is treated [8]. A large proportion of the untreated nets are purchased in the private sector; 46% are made by tailors [44]. These facts underscore the importance of the on-going discussions in Mali surrounding the promotion of insecticide-treated nets (ITNs) and treatment kits. While the success of the promotion of bed net treatment kits will depend on the development of creative and inexpensive educational and promotional activities, improving the capacity to monitor and evaluate the use of treatment kits is crucial. Moreover, observed heterogeneity in the risk of a presumptive malaria diagnosis by CSCOM underscores the importance of malaria studies conducted at the local level in urban areas, which could facilitate the implementation of locally targeted interventions [14-16].

This study entails a number of limitations. First, the consultation data is an endogenously selected sample (choice-based sample) as it provides information only on children under age five who sought health care and received a consultation at a CSCOM. It is possible that children who chose not to attend the CSCOM may differ in important ways from those who did. However, the analysis aimed to describe the secular trend in malaria consultation rates only among health-care seeking children. Second, due to the nature of information recorded in the consultation registries, it is impossible to determine whether the same child had multiple records over time. To accommodate this, in the multilevel analysis, the individual unit of observation was the CSCOM and not children. Third, during the rainy season, when malaria transmission is greatest, household revenue is likely to decrease. This could potentially result in less health care seeking and a greater use of traditional medicine [45]. Therefore, it is possible that the estimates of presumptive malaria based on consultation registries may underestimate the total malaria burden during the rainy season. However, it was assumed that any underestimation of the total malaria burden during the rainy season was consistent throughout the study period as there were no reported significant economic downturns during the study years. Finally, there is the question of the validity of using a presumptive malaria diagnosis, in the absence of control variables, to demonstrate intervention efficacy. While the variable of the study cannot be changed, the question of control variables speaks to the impossibility of controlling for all possible factors reducing transmission (e.g., urban growth *per se *can contribute to a reduction in malaria transmission) [46]. In addition, identifying independent effects of multiple interventions would require different data collected under a very specific design. Yet, this study showed that there is a correlation between the bed net treatment kit intervention and the decline in the presumptive malaria consultation rate; this is a correlation not found for the other two interventions.

In conclusion, with the increasing awareness that infectious diseases, particularly malaria, impose an enormous burden on developing countries, the use of appropriate control interventions is essential. The MoH has set a target year of 2011 to reduce the national level of malaria morbidity by 50% as compared to year 2000 levels, with a particular focus on children under five [47]. Results presented in this study suggest that the burden of urban malaria in children under age five is still significant. This finding becomes even more relevant considering the fast growth of urban areas in Africa [48,49]. Despite recent declines in malaria consultation rates, interventions targeted to reduce malaria transmission among children in urban areas are needed, particularly regarding the expansion of coverage and increased usage of ITNs.

## Conflicts of interest

The authors declare that they have no competing interests.

## Authors' contributions

ARW, SD, and MCC participated in the study design. ARW, SD and BT participated in study implementation. ARW collected the data. ARW analyzed the data and interpreted the results under the supervision of MCC. ARW and MCC wrote the paper with input and editing from SD and BT. All authors read and approved the final version of the paper.
